# Impact of Iron Mining Activity on the Endophytic Fungal Community of *Aspilia grazielae*

**DOI:** 10.3390/jof9060632

**Published:** 2023-05-30

**Authors:** Carlos Eduardo Weirich, Maria Rita Marques, Alinne Pereira de Castro, Beatriz Assumpção Benitez, Fabio de Oliveira Roque, Clarice Rossato Marchetti, Amanda Dal’Ongaro Rodrigues, Dênis Pires de Lima, Edson dos Anjos dos Santos

**Affiliations:** 1 Laboratório de Bioquímica Geral e de Microrganismos, Instituto de Biociências, Universidade Federal de Mato Grosso do Sul, Cidade Universitária, Campo Grande 79070-900, MS, Brazil; carlosweirich@yahoo.com.br (C.E.W.); marquesmariarita@gmail.com (M.R.M.); crmarchetti@gmail.com (C.R.M.); amandadalongaro@hotmail.com (A.D.R.); 2 Departamento de Biotecnologia, Universidade Católica Dom Bosco, Campo Grande 79117-900, MS, Brazilbiahs123@hotmail.com (B.A.B.); 3 Programa de Pós-Graduação em Ecologia e Conservação, Instituto de Biociências, Universidade Federal de Mato Grosso do Sul, Campo Grande 79070-900, MS, Brazil; roque.eco@gmail.com; 4 Centre for Tropical Environmental and Sustainability Science (TESS), James Cook University, Cairns, QLD 4878, Australia; 5 Laboratório de Pesquisa 4, Instituto de Química, Universidade Federal de Mato Grosso do Sul, Campo Grande 79070-900, MS, Brazil; dpireslima@gmail.com

**Keywords:** *Aspilia grazielae*, fungal endophytes, mining activities

## Abstract

*Aspilia grazielae* (J. U. Santos) is an endemic plant species in Morro do Urucum in the Pantanal wetland (Brazil). *A. grazielae* is used for the restoration of areas impacted by iron mining activities. This study evaluates the diversity (composition, value and abundance) of endophytic fungal communities, considering parts of the plant and soil condition. The leaves and roots of *A. grazielae* were collected from native vegetation areas (NVA) and recovery areas (RCA) in Morro do Urucum. Illumina sequencing technology was used to investigate variation in endophytic fungal biodiversity. The operational taxonomic units detected in NVA ranged from 183 to 263 (leaf) and 115 to 285 (root), while RCA samples ranged from 200 to 282 (leaf) and 156 to 348 (root). Ascomycota phylum was the most common species among all plant samples. The most significant classes identified were Lecanoromycetes and Dothideomycetes that differed significantly (*p* ≤ 0.05) according to their plant hosts and soil stress. The relative abundance of *Pestalotiopsis* (Sordariomycetes class) and *Stereocaulon* (Lecanoromycetes class) genera was influenced by the iron mining activities according to the leaf samples analysed. However, the abundance and wealth of endophytic fungal communities in *A. grazielae* from RCA were evidence that could explain their high resilience to environmental disturbances and the source-sink dynamics of fungal propagules.

## 1. Introduction

The Brazilian Pantanal is the largest continuous wetland in the world [1]. Anthropogenic activities threaten its biodiversity and ecological services [1,2]. In the coming years, agricultural and mining activities may lead to negative ecological impacts [2,3]. In addition, huge forest fires devastated almost one third of the Pantanal in 2020 [4]. The Pantanal is losing biodiversity that has not yet been described. For example, most fungi and bacteria associated with plants are barely known in the region [5,6]. Among these communities, the fungi communities associated with plants are particularly important because they have a strong symbiotic relationship that supports plant growth [7,8] helping to defend them against pathogens [9,10]. Fungi are instrumental in carbon and nitrogen cycling and participate in the bioremediation of xenobiotics [11,12,13]. Accordingly, learning about the relationship between fungal communities and plant species in the Pantanal is fundamental to understanding how it is influenced and changed by land use and management. This is an urgent task for the conservation and sustainable use of biodiversity in this region.

Endophytic fungal communities are ubiquitous and many of them are essential for the adaptation of their host plants to specific environments, such as recovery areas from mining activities. These microorganisms grow asymptomatically in root cells, stems and leaves and are significant mutualists, increasing the uptake of nutrients by physical and chemical means [7,14]. Studies on the diversity of endophytic fungi have shown that a single plant can be colonized by more than 1000 microbial species, and most of them cannot be cultivated [12]. To partially assist in the control of plant pests and pathogens, the endosphere has become the subject of intensive research [10].

The microbial endophytic diversity in plants that inhabit regions with harsh environmental conditions can help in understanding the adaptation of these plants to such ecosystems [15,16]. The vegetation restoration of areas exploited for mining is a challenge due to adverse conditions of the soil, such as the low concentration of organic material and alterations in the physical-chemical properties of soil [17,18]. Endophytic fungi from mining areas have a strong symbiotic relationship with plants, as they are associated with stimulating and maintaining healthy plant growth [10,19,20].

Studies have shown that there is a dynamic correlation between the composition and diversity of the microbial community, biotic factors such as vegetation cover and abiotic factors such as soil quality [21,22]. The mycorrhizal fungi play complex functional roles in the adaptability of plants in metalliferous environments, considerably contributing to the growth and development of their hosts in inhospitable environments [20]. Kalu et al., (2021) found that the influence of root exudation on the composition of the rhizosphere microbial community is a critical factor for the growth and adaptability of the *P. australis* species to a metal-laden ecological niche, with acidic pH in AMD (Acid Mine Drainage) [23]. 

The rare plant species *Aspilia grazielae* (Asteraceae) is a tree-like shrub endemic to the Brazilian flora of the Pantanal [24,25]. The species is threatened by the ongoing mining activities in the Pantanal, which leads to a continuous degradation of the quality of its habitat [26]. Studies on *A. grazielae* are limited to checklists and reports on its distribution [25], and it is on the Red List of threatened and endangered species in Brazil [27]. However, *A. grazielae* has been used by the mining company Vale S.A. to restore degraded areas [28,29]. There is an urgent need to address the lack of biodiversity knowledge of tropical plant species (especially the threatened and endangered ones) as well as of fungi communities. Therefore, in this study, we evaluated, for the first time, taxonomic facets of the diversity of endophytic fungal communities (their value, abundance and diversity) in leaves and roots of the plant *A. grazielae* growing in two areas in Morro do Urucum (Pantanal Wetland, Brazil) through different levels of anthropogenic intervention in the soil and vegetation cover. Although descriptive, our study contributed to solving the tropical biodiversity puzzle which is critical for expanding and improving the use of DNA in monitoring, conservation, restoration and other applied areas.

## 2. Materials and Methods

### 2.1. Study Area

**Figure 1:**
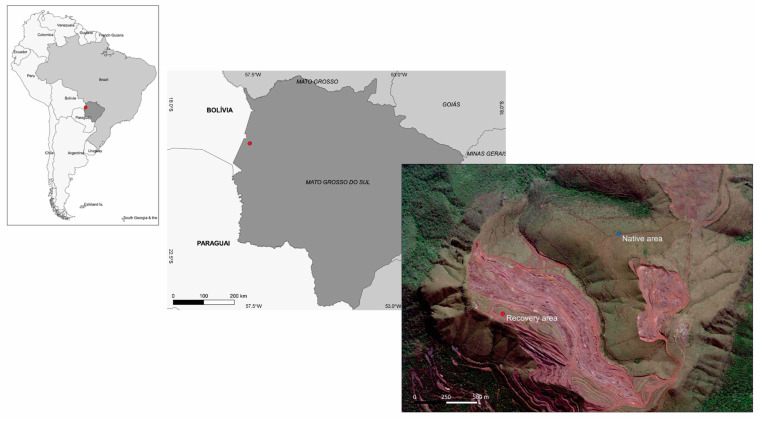
Map of the two areas where *A. grazielae* samples were collected, in Morro do Urucum (Pantanal biome), State of Mato Grosso do Sul, Brazil.

The Upper Paraguay Basin (BAP) is a transitional region between the major biomes of South America: Pantanal, Amazon, Atlantic Forest, Cerrado, Chaco, Bosque Chiquitano and the remnants of the Caatinga [30]. The vegetation is highly variable and consists mainly of grasslands, seasonal deciduous and semi-deciduous forests, rock formations on the hills, laterite vegetation, flooded riparian forests of the Paraguay River and flooded forests associated with black bays [31]. The studies were carried out in Morro do Urucum (Pantanal biome), located to the south of the urban areas of Corumbá and Ladário in the state of Mato Grosso do Sul, at coordinates 19°18′44.2″ S, 57°61′125 W″ (recovery area—RCA) and 19°17′85.2″ S 57°60′22.4″ W (native vegetation area—NVA) (Figure 1).

The altitude varies between 500 and 1000 m and is characterized by the presence of rock conglomerates of granites, gneisses, limestones and micaxites [32]. The climate is classified as metamorphic-tropical, with an annual average temperature of 25.1 °C and annual relative humidity of 76.80%. The average rainfall is 1070 mm, with the rainy season occurring between November and March, while the driest periods are between June and August. In the studied region, the main soil types are eutrophic lithosols and cambisols. The region is commercially exploited for open pit mining of iron and manganese. Iron is extracted with the complete removal of vegetation and soil [29].

### 2.2. Plant Collection and Soil Samples

The plants used in the recovery areas came from the same region of study in the Pantanal biome, which is the Morro do Urucum, Corumbá, Brazil. The mining company Vale S.A. obtained seedlings from cutting or seed germination of adult plants in native areas. The soil used to cultivate seedlings was the same as native areas. These seedlings are grown in field conditions and then replanted in the recovery areas. Reforestation management is as natural as possible, without control of variables such as light, humidity or temperature. The soil did not receive any kind of pre-treatment.

The plants and soil samples were collected in March 2015. Five individuals of *A. grazielae* were selected in each area studied. We collected branches and roots of each individual within a radius of 100 m between plants to reduce intraspecific variation. The plants had a healthy appearance and showed no obvious disease symptoms. Samples were placed in sterile plastic bags, labelled by area, placed in a thermal box, and transported to the laboratory. One voucher specimen (UFMS nº 59308) was deposited in the herbarium of Campo Grande Mato Grosso do Sul (CGMS) after its botanical identification.

Soil samples from the two areas studied were collected at a depth of 0–20 cm to characterize the physicochemical properties, with a total of five samples from each area (Tables S1 and S2). All samples were identified, immediately stored in sterile plastic bags and transported to the laboratory in a thermobox. A 2 mm portion of the soil was sieved and homogenized for chemical analysis. The physicochemical properties of the soil were determined according to protocols [33,34].

### 2.3. Pre-Treatment of the Plant Samples

Each plant was divided into leaves and roots and rinsed under running water to eliminate soil and dust before complete genomic DNA extraction. The sample was then placed in sterile plastic bags, labelled and stored at −18 °C until DNA was extracted. Epiphytic microorganisms were eliminated by superficial cleaning with sterile water for 20 s on the leaves and 10 cm segments of roots of each plant at random. They were then soaked in NaOCl 3% (0.4 mM) for 10 min, which would be enough to cause considerable damage to the remaining DNA structures, making their analysis impossible [35,36]. The samples were then submerged in 70% ethanol for 30 s, washed three times with sterile water and dried under aseptic condition [37,38]. Aliquots of the water from the final rinse solutions of each sample were plated in BDA media to confirm the surface disinfection. Cut into 0.5 cm pieces, the leaves and roots were macerated in a porcelain crucible with the addition of liquid nitrogen.

### 2.4. Extraction of Genomic DNA, PCR Amplification and ITS rDNA Sequencing

DNA genome extraction from roots and leaf samples from *A. grazielae* was adapted using the reported procedure [39]. Illumina MiSeq was performed for the twenty samples. Internal transcribed spacer (ITS) sequencing for fungal community analysis followed a previous protocol [39]. Isolated root and leaf sample DNAs were stored below −20 °C until PCR amplification. The full primers constructed were long because for the 5.8S and fun primer there are included: RC of Illumina 3′ Adapter (CAAGCAGAAGACGGCATACGAGAT) plus Golay Barcode (CGTACCAGATCC) plus Reverse Primer Pad (AGTCAGTCAG) plus Reverse Primer Linker (GG) plus 5.8S_Fun (AACTTTYRRCAAYGGATCWCT). Additionally, for ITS4_Fun primer there are included: 5′ Illumina Adapter (AATGATACGGCGACCACCGAGATCTACAC) plus Primer Pad (TATGGTAATT) plus Primer Linker (AA) plus ITS4_Fun (AGCCTCCGCTTATTGATATGCTTAART) [40]. Paired-end sequencing (2 × 250 bp) was accomplished using Illumina MiSeq sequencer (Illumina, San Diego, CA, USA).

Raw sequence readings were first filtered according to standard parameters. The overlapping clean readings were assembled into consensus sequences (namely tags) using the Fast Length Adjustment of Short reads, version 1.2.11 (http://ccb.jhu.edu/software/FLASH, accessed on 12 August 2019) program. The tags were further clustered into operational taxonomic units (OTUs) with a sequence identity of 97% as the threshold using UPARSE (http://www.drive5.com/uparse, accessed on 12 August 2019), and chimeras were filtered out using UCHIME, version 4.2.40 (http://drive5.com/uchime, accessed on 12 August 2019). The OTUs were taxonomically classified using the Ribosomal Database Project Classifier, version 2.2 (http://rdp.cme.msu.edu, accessed on 12 August 2019), referencing the UNITE ITS database (https://unite.ut.ee, accessed on 12 August 2019), using 0.6 confidence values as the cut-off [41]. The Illumina sequencing data obtained in these experiments are publicly available in the NCBI under BioProject number PRJNA563914 (https://www.ncbi.nlm.nih.gov/bioproject/PRJNA563914/, accessed on 4 September 2019).

### 2.5. Statistical Analysis

We evaluated our sampling effort by building a rarefaction curve of the observed OTU number of fungal endophytes against the number of leaf and root samples at each study area. To compare values of the number of OTUs, we used Shannon, Simpson, and Chao1 Diversity. The indexes between roots and leaves from native and recovery areas were calculated using a *t*-test (equal variance) with type two-sided using the Statistical Analysis of Metagenomic Profiles (STAMP) software version 2.0.0 [42].

A Venn diagram was created to depict the spread of the fungal population in *A. grazielae* root and leaf samples. The effects of regions, leaves, and roots on the patterns of fungal composition were investigated using a principal coordinate analysis (PCoA) based on the Bray–Curtis dissimilarity matrix among samples (Monte-Carlo, 999 permutations).

PCA was used to perform multivariate data analysis. The data were previously self-scaled to adjust for discrepancies in the order of magnitude in the variables, as they are physical and chemical characteristics, and all variables were given the same weight in the PCA models. For this study, a PCA was performed using all the samples and physical and chemical attributes of the two areas, to promote an investigation of the influence of land use in the two evaluated areas (NVA × RCA).

The taxonomic differences among multiple samples were calculated by ANOVA followed by a post hoc Turkey test with *p* ≤0.05 using the software GraphPad Prism 8.0 and STAMP 2.0.0 [42].

## 3. Results

### 3.1. Soil Analysis

**Figure 2:**
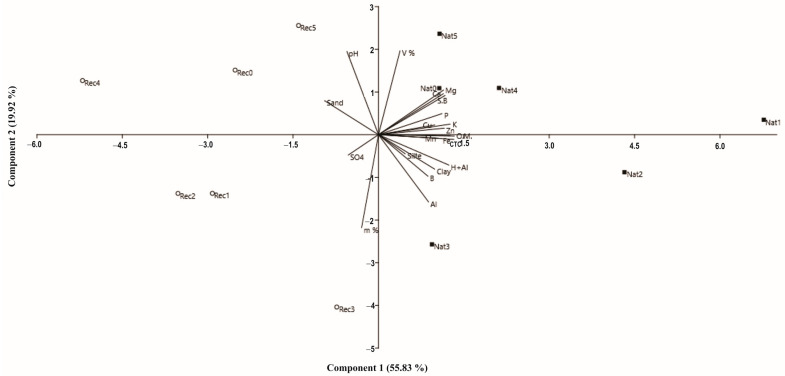
Graph of PC1 × PC2 scores and weights of physical attributes and soil fertility, labelled by land use: ○Nat (areas with native vegetation—NVA) and ■Rec (recovery area—RCA). O.M.—organic matter, pH—hydrogen potential, S.B—sum of bases, CTC—cation exchange capacity, V—base saturation, m—aluminium saturation, H + Al—exchangeable acidity, P—phosphorus, K—potassium, Ca—calcium, Mg—magnesium, Al—aluminium, B—boron, Cu—copper, Fe—iron, Zn—zinc, Mn—manganese, SO4—sulphates, Clay, Sand, Silte.

A Principal Component Analysis (PCA) was performed on the data matrix, which consisted of 21 variables from the two study areas to reduce the dimensionality of such data sets. The data were standardized, as there are large differences in the magnitude of the individual variables (Figure 2).

A PCA reveals clear differences in the physical-chemical characteristics and properties of the soil between the native vegetation area (NVA) and root from the recovery area (RCA). The first component explains 55.83% of the data variability, while the second explains 19.92%. The results revealed that soil samples from the NVA have a high concentration of clay and organic matter and low concentration of sand, whereas soils from RCA have the opposite trend. Another negative correlation can be observed between the pH and aluminium between the two areas: while the pH is higher in RCA than in NVA, the concentration of Al is lower in RCA than in NVA. Soils rich in organic matter, such as those found in the NVA, correlated positively with Cation Exchange Capacity (CTC), total acidity (H + Al), potassium (K) and iron (Fe).

### 3.2. Community Diversity

**Figure 3:**
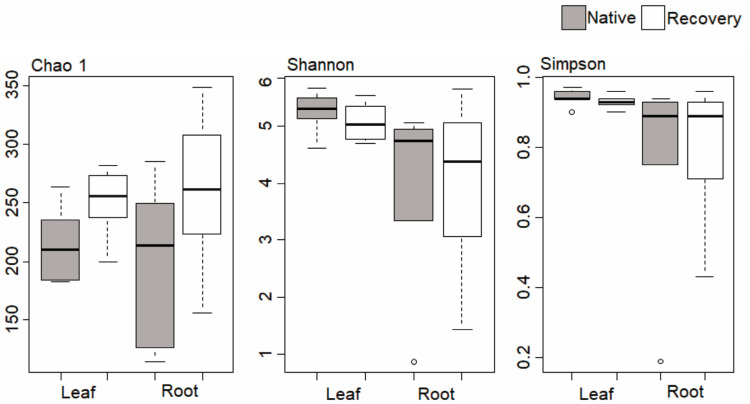
Chao 1 wealth index and Shannon and Simpson diversity for samples of leaves and roots from native and recovery areas. The mean value (line) and the confidence interval (dotted) in each group are also illustrated.

After data normalization, the total number of ITS region sequences was 1,136,278, corresponding to 4611 operational taxonomic units (OTUs). The number of OTUs detected in individual samples ranged from 183 to 263 in NVA leaves and from 200 to 282 in RCA leaves. The values for roots ranged from 115 to 285 OTUs in NVA and from 156 to 348 in RCA. According to OTU data, Shannon, Simpson, and Chao1 Diversity Index estimates revealed that there was no significant difference between the evaluated areas when comparing the leaves and roots (Figure 3).

**Figure 4:**
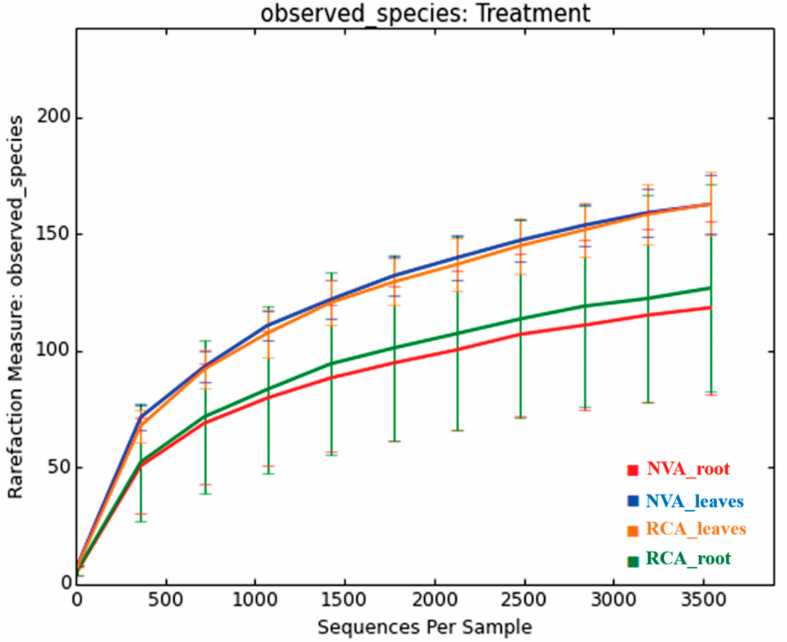
Rarefaction curve of the observed operational taxonomic units (OUTs) number of fungal endophytes species against the number of samples (5 replicates per sample) in each study area. Samples were composed of roots from native vegetation area (NVA), root from recovery area (RCA), leaf from NVA and leaf from RCA.

The rarefaction curves of all *A. grazielae* samples tended to approximate the saturation plateau (Figure 4), which confirms a sampling sufficiency of the communities under study.

**Figure 5:**
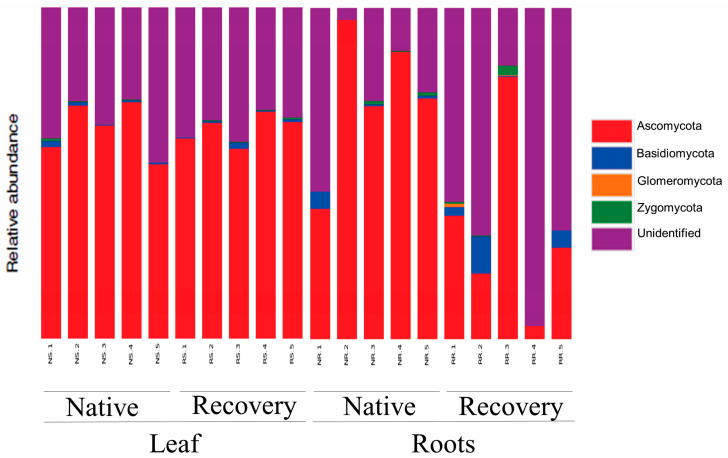
Phylum-level taxonomic affiliation of internal transcribed spacer (ITS) sequences of endophytic fungi living in leaves and roots of *A. grazielae*, growing in native soil or mining soil under the recovery of transition region between Cerrado and Pantanal biomes in Brazil.

The OTUs were assigned to 4 fungal phyla, 14 classes, 41 orders, 79 families and 176 genera for root samples and 3 phyla, 15 classes, 35 orders, 66 families and 137 genera for leaf samples. Ascomycota members were the most common among all plant samples with 63.3% and 53.3% of relative abundance (RA), followed by Basidiomycota with 0.8% and 2.6% RA for leaf samples and roots, respectively, while Zygomycota (0.2% and 0.6%) and Glomeromycota (0.0% and 0.1%) were rare and accidental (Figure 5).

**Figure 6:**
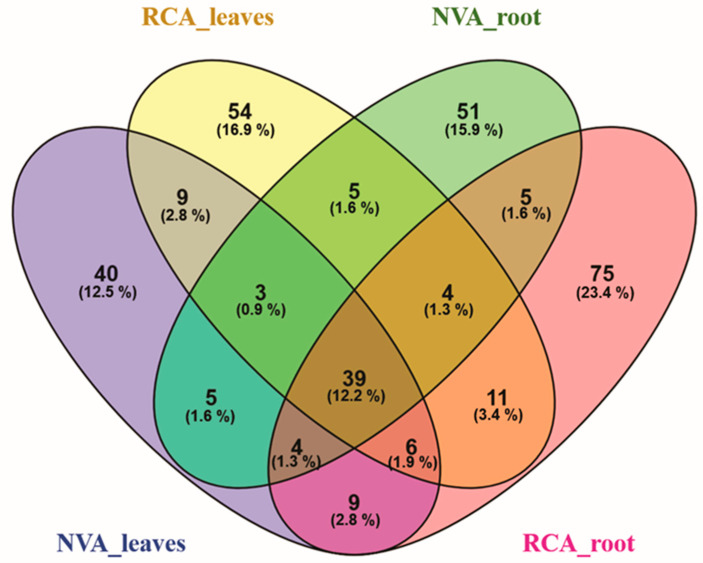
Venn diagram showing the degree of overlap of operational taxonomic units (OTUs) fungi between leaves and roots of *A. grazielae* collected in native vegetation area (NVA) and in recovery area (RCA).

The specific fungal community was higher in root samples of RCA with 23.4% than in NVA with 15.9%, and 1.6% of OTUs were shared between the areas (Figure 6). The variation of the OTUs was 16.9% in leaves from RCA and 12.5% in leaves from NVA, and 2.8% of OTUs were shared between areas.

**Figure 7:**
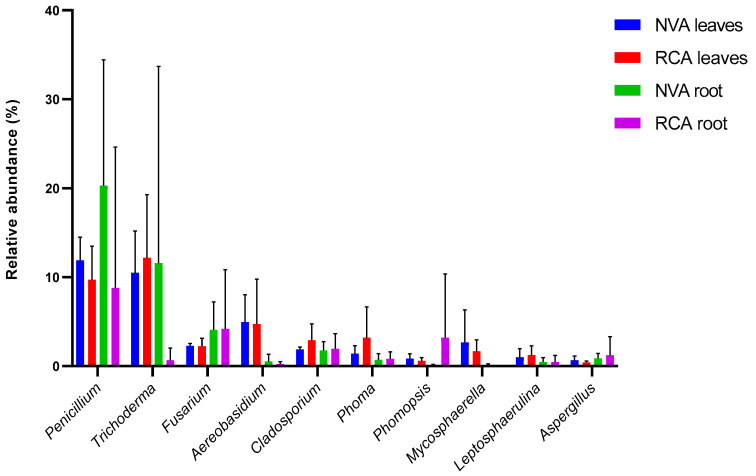
Relative abundance (RA) ≥ 3.0% of endophytic fungal genera in samples of leaves and roots of *Aspillia grazielae* from the native area (NVA) and from the recovery area (RCA). The *p*-value for statistical significance was defined as *p* ≤ 0.05.

The Relative Abundance (RA) showed some differences, in percentage terms, between the two areas and between the analysed tissues (Figure 7). *Trichoderma* and *Penicillium* were the most common genera in NVA roots, followed by *Fusarium*. *Penicillium* and *Phomopsis* were more common in RCA roots. The leaves genera profile did not show significant differences (*p* ≤ 0.05) between the two areas, i.e., NVA and RCA (Figure 7).

**Figure 8:**
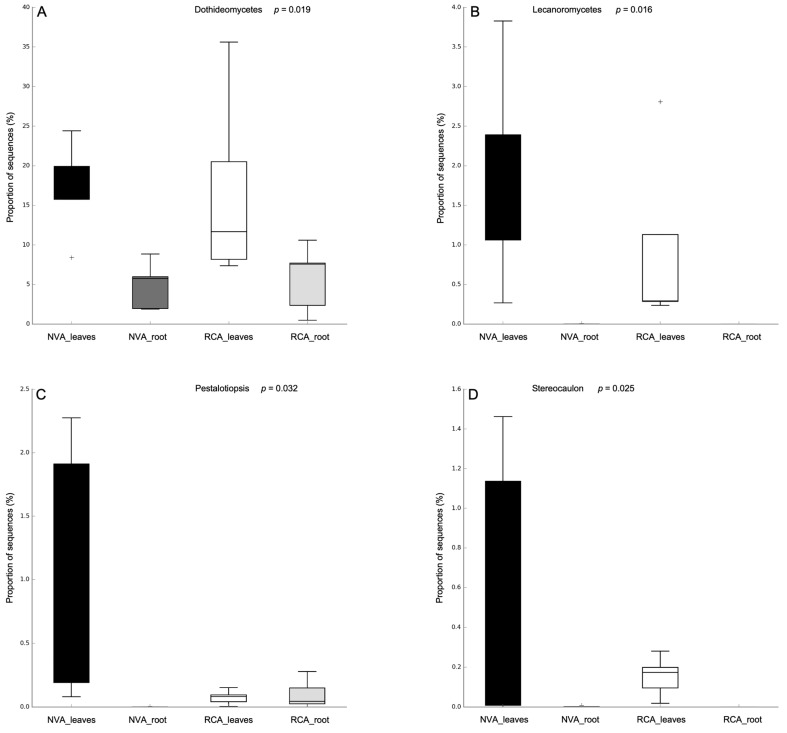
The fungal relative abundance (RA) profile by proportion of sequences (%) in leaves and roots of *A. grazielae* collected from native vegetation area (NVA) and recovery area (RCA): Dothideomycetes class (**A**), Lecanoromycetes class (**B**), *Pestalotiopsis* genus (**C**) and *Stereocaulon* genus (**D**). The *p*-value for statistical significance was defined as *p* ≤ 0.05.

The proportion of sequences (%) of the main fungi from leaves and roots of *A. grazielae* collected from NVA and RCA are shown in box plots (Figure 8). In the proportion of sequences index (Figure 8), the most significant classes identified are Lecanoromycetes and Dothideomycetes (Ascomycota) (Figure 8A,B), which differed significantly according to their plant hosts and soil stress. Among these, significant presence of Lecanoromycetes (Figure 8B) was not identified in root samples in both NVA and RCA. In contrast, the RA of the Lecanoromycetes class increased in leaf samples in NVA (NVA_leaves). Sequences associated with the Lecanoromycetes class, including the *Stereocaulon* genus (Figure 8D), presented the same pattern of presence. However, they were more present in NVA_leaves than in RCA_leaves, agreeing with the Lecanoromycetes class pattern. The Dothideomycetes class (Figure 8A), on the other hand, showed higher RA in sample leaves from the RCA when compared with samples from NVA. Considering the roots, RCA samples showed a small rise (RCA_root). This class had a considerably higher presence in the whole RCA. In addition, the *Pestalotiopsis* genus presented a huge decrease in RA comparing NVA_leaves samples to RCA_leaves samples. Considering the roots, there was a significant increase in RCA samples when compared with NVA samples (Figure 8C).

**Figure 9:**
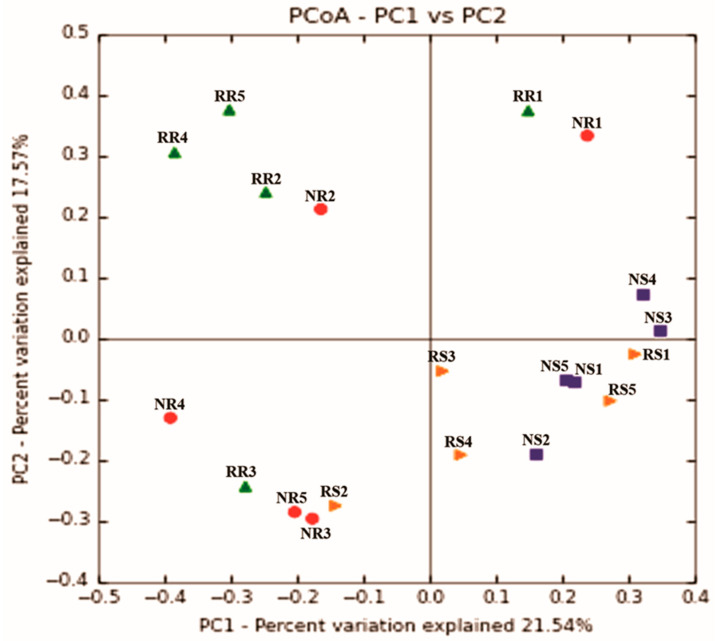
Principal Coordinate Analysis (PCoA), based on a Bray–Curtis beta diversity matrix, describing the structure of the fungal community associated with *A. grazielae* roots and leaves from native vegetation area (NVA) and recovery area (RCA). The fungal endophytic communities of each area and each part (root and leaves) of the plant are indicated by geometric symbols with different colours: ● (NR: root of NVA); ▲ (RR: root of RCA); ■ (NS: leaves of NVA); ► (RS: leaves of RCA).

The Principal Coordinate Analysis (PCoA) showed that the overall fungal microbiota at RCA and NVA did not differ between groups (ANOSIM *p* > 0.05; Figure 9). However, there was a significant effect of sampling samples (root vs. leaf) on the overall fungal community composition (*p* < 0.002; Figure 9).

## 4. Discussion

### 4.1. Revealing the Diversity of Endophytic Fungi in Different Plant Tissues

In the Pantanal wetland, a limited number of plant species have had their endophytic fungal communities studied [5]. In this research, we have described the diversity of endophytic fungal communities associated with *A. grazielae* leaves and roots. In the tropical region, plant tissues support distinct endophytic fungal communities [43]. Although the number of taxa per host varies greatly between plant species, some authors estimate that there are 30 endophytic fungal species on average per plant [44,45]. Root systems typically have more species than leaves, and these systems are dominated by various fungal species. Mining sites have environmental challenges for the establishment of plant species, and the association of symbiotic fungi to the roots is considered a decisive role for their survival and maintenance [9].

As expected, there were more *A. grazielae* readings in root tissues (980,160 readings) than in leaves (228,118 readings). This pattern could be explained by the fact that fungi associated with roots are more opportunistic, and several species living in soil litter colonize plant roots [46].

Ascomycota, which is the most common in roots (53.3%) and leaf samples (63.3%), is a dominant phylum in many plant species and appears to be a general feature of the endophytic microbiota associated with tropical plants [47,48]. Despite having a much lower frequency, operational taxonomic units (OTUs) from the Zygomycota and Basidiomycota phyla were found in *A. grazielae* tissue samples from both areas. Wehner et al. (2014) studied the fungal community associated with Asteraceae roots and discovered that 18.3 percent of the sequences belonged to the Basidiomycota phylum and 7.2 percent to the Glomeromycota phylum [49]. The relationships between fungal dominance and plant species differ depending on taxonomic group, study area and spatial scale [50]. More data, however, are required to provide substantial support for any generalization about the factors that regulate dominance in these communities.

### 4.2. Diversity of Endophytic Fungi in A. grazielae Considering Land Use, Soil and Vegetation

The NVA and RCA have metallic soil, rich in iron and manganese. Native plant species and consequently the associated endophytic microbiota have been adapting to this metalliferous environment over time, which turns these communities into potential phytoremediators [51,52].

The relative abundance (RA) of the endophytic genera in *A. grazielae* from both native (NVA) and recovery (RCA) areas did not differ significantly, considering the most abundant genera. Some taxa seem to be associated with the conditions generated by the restoration, and they may even have a bioremediation role. For example, *Trichoderma* [53], *Penicillium* [54], *Fusarium* [55] and *Phomopsis* [54].

The genus *Trichoderma* is extensively studied as a mycoparasite of phytopathogenic fungi, playing a key role in the growth and development of its host plants [56]. Moreover, some species act as bioremediators of soil pollutants such as arsenic [57], cadmium [47] and lead [57].

On the other hand, the classes identified as Lecanoromycetes and Dothideomycetes and the genera *Stereocaulon* and *Pestalotiopsis* showed significant differences in RA comparing the NVA and RCA.

Growing evidence suggests that the secondary metabolites are found in the main fungi identified in this study. Both the Dothiomycetes, Lecanoromycetes classes and the *Pestalotiopsis* genus (Sordariomycetes class) are known as a huge source of natural compounds of biotechnological interest, improving plant tolerance to biotic and abiotic stress [58,59,60]. The presence of the Dothiomycetes class in all samples can be related to their high tolerance to heavy metals, compared to other fungal classes, already described by Sim et al., (2018), which may be a competitive advantage in plant colonization [61]. More recently, it was revealed that the endophytic *Aerobasidium* (Dothideomycetes class) in *Cucumis sativus* plants could improve the soil metal tolerance during contamination by regulating soil enzymatic activities, reducing the metal uptake and improving the plant antioxidant system [62]. The authors emphasize that the endophytic *Aerobasidium* is a promising phytoremediation agent for crops growing in Pb and Cd polluted soil [62]. Additionally, in the NVA leaf samples, there was a higher RA of the Lecanoromycetes class, and the *Pestalotiopsis* and *Stereocaulon* genera were lower in the RCA leaf samples, indicating that these fungal groups were highly influenced by the iron mining activities.

Abiotic and biotic factors govern microbial community organization in the rhizosphere [63,64]. Soil has a complex influence on the formation of bacterial and mycorrhizal fungal communities in the rhizosphere [65]. Complex physicochemical properties of soil influence plant physiology, and root exudate patterns influence rhizosphere microbiota composition [14,66,67]. Our findings show that the soil characteristics differ between RCA and NVA, as expected. The RCA soil is predominantly sandy, with higher pH, and the concentration of Al and organic matter is lower, which may explain the high abundance of Ascomycota, a very generalist and tolerant group of fungi [68], in plant root systems in this area. Accordingly, soil nutrients can influence microbiome characteristics [69,70]. The concentration of organic matter in the NVA may have had a significant impact on soil macro and micronutrients. Because it is associated with greater moisture retention [71], organic matter serves as an important source of nutrition during the decomposition process, which influences the microbial communities in the evaluated areas.

Furthermore, anthropogenic changes such as mineral extraction, removal of vegetation and the resulting loss of biodiversity of local flora may have contributed to the difference in the composition of the root endophyte communities between the two areas. These facts resulted in the selection of genera that are better suited to a particular soil profile. For example, the *Phomopsis* genus had a relative abundance (RA) greater than 1% in the roots of the RCA, whereas the *Trichoderma* genus had a lower RA in this area compared to the NVA.

Endophyte fungi were more abundant and diverse in the RCA than in the NVA. Firstly, the sources of propagules from regional species groups influence local community dynamics [14,72]. For example, when a resource becomes available on a new site, the first taxa to colonize it have a significant competitive advantage and may exclude later arrivals. Because the RCA has been subjected to numerous colonization events, and its sandy soil is more prone to being washed away by storms, fungi communities may be more abundant and diverse because of the increased spore availability in a continuous process of colonization and re-colonization. The analysis of the endophytic fungal community structure among *A. grazielae* individuals reveals a higher RA of endophytic fungi among the leaf samples and roots of the RCA, with few OTUs shared between both areas. In contrast to the plant stabilized NVA endophyte communities, the anthropogenic process of mining may have favoured the colonization of new endophytes in *A. grazielae* from RCA.

According to Liu et al., (2022) a selective evolution of fungal species associated with hosts involved in the resistance or transformation of metals or metalloids may occur in a metalliferous ecosphere [52]. Understanding the structure of native fungal communities, as well as the hosts that maintain the diversity of their microbial communities in intensive mining areas, can provide important information related to the adaptive processes of these genera/species to these metalliferous environments. Such findings can be very useful tools in remediation processes in mining areas.

## 5. Limitations, Implications and Perspectives

Although our study represents a clear advance for the mapping of fungi and plants in the Pantanal, it is important to recognize limitations and challenges. First, most of the OTUs detected in our study reveal the great challenge of identifying fungi in the studied region. Isolation of varieties and descriptions of species is still a major obstacle to knowledge of these interactions. Second, in our study, we assumed that most of the fungi were detected in endophytes. However, we still need experiments to assess the degree of dependency between species. Third, the understanding of the ecological mechanisms that operate the dynamics of colonization and coexistence of fungi in plants in complex landscapes such as those evaluated in our study requires more robust experimental designs and, if possible, manipulative ones to test the hypotheses raised in our work. In addition, we highlight the need for more studies on the role of fungi in plant health, particularly *A. grazielae*.

In an applied perspective, it is important to highlight that understanding the diversity of fungi communities in plants is the first and most important step towards using them as bioindicators of the recovery process or to aid in the acceleration of the recovery process in mining restoration initiatives.

The use of *A. grazielae* plants can be an important tool in the management of areas polluted by mining, due to its resilience to high concentrations of metals such as iron and manganese. We indicate here that the endophytic fungal community of this plant plays a very important role in this resistance process, and future studies of the isolated genera may be useful for bioremediation work.

## Data Availability

The draft genome sequences of samples are available on NCBI (BioSample) databases through the accession numbers SRX6806294 to Recovery_Root sample (https://www.ncbi.nlm.nih.gov/sra/SRX6806294[accn], accessed on 31 December 2019), SRX6806293 to Native_Root sample (https://www.ncbi.nlm.nih.gov/sra/SRX6806293[accn], accessed on 31 December 2019), SRX6806292 to Recovery_Sheet sample (https://www.ncbi.nlm.nih.gov/sra/SRX6806292[accn], accessed on 31 December 2019), SRX6806291 to Native_Sheet sample (https://www.ncbi.nlm.nih.gov/sra/SRX6806291[accn], accessed on 31 December 2019).

## Supplementary Materials

The following supporting information can be downloaded at: https://www.mdpi.com/article/10.3390/jof9060632/s1, Table S1: Physicochemical properties of soil fertility in samples from the native vegetation area (NVA) and the recovery area (RCA); Table S2: Relative abundance of OTUs taxa (%) ≥0.5 found in plant tissues (roots and leaves) of Aspillia grazielae (Ascomycota Phylum).
