# Supplementary material for: Impact of Iron Mining Activity on the Endophytic Fungal Community of Aspilia grazielae
Source: J Fungi (Basel). 2023 May 30;9(6):632. doi: 10.3390/jof9060632 (PMC10303068; doi:10.3390/jof9060632)
Supplement: Supplementary file 1 [file jof-09-00632-s001.zip › jof-2246016-supplementary.pdf]

Journal of *Fungi*

MDPI

Table S1. Physicochemical properties of soil fertility in samples from the native vegetation area (NVA) and the recovery area (RCA).

Parameters	NVA						RCA					
	NVA0	NVA1	NVA2	NVA3	NVA4	NVA5	RCA0	RCA1	RCA2	RCA3	RCA4	RCA5
O.M. mg/dm^3	54.2	110.9	75.2	55.6	60.1	48.4	16.3	15.7	14.2	34.5	5.3	27.3
pH	4.5	4.2	4.1	4	4.5	4.7	4.7	4.3	4.4	4.1	4.6	4.9
S.B mmolc/dm^3	37	62	37	14	46	48	16	6	4	7	5	15
CTC mmolc/dm^3	107	219	171	102	119	102	43	37	40	100	23	78
V %	35	28	22	14	38	47	37	17	10	7	20	33
m %	16	21	30	54	14	6	13	44	51	72	30	3
H+Al mmolc/dm^3	70	158	133	88	73	54	27	30	36	93	19	37
P mg/dm^3	5	10	9	5	6	3	4	3	2	2	2	7
K mmol/dm^3	2.6	4.4	3.2	2.3	4	2.7	1.4	1.5	0.6	2.3	0.4	2.4
Ca mmolc/dm^3	28	44	28	9	32	39	11	4	3	4	3	10
Mg mmolc/dm^3	7	13	6	3	9	7	3	1	1	1	1	6
Al mmolc/dm^3	7	16	16	17	7	3	4	5	4	17	2	1
B mg/dm^3	0.5	0.6	0.5	0.6	0.6	0.5	0.4	0.5	0.4	0.6	0.2	0.5
Cu mg/dm^3	1	1	1.3	0.9	0.4	1.2	0.3	0.4	0.6	0.3	0.1	0.5
Fe mg/dm^3	74.6	124.3	104.5	65.5	46.3	56.5	23.3	15.5	16.5	32.6	21.1	22.3
Zn mg/dm^3	0.5	1.1	0.9	0.6	0.4	0.3	0.4	0.2	0.2	0.2	0.2	0.5
Mn mg/dm^3	22.9	46.6	48.6	22.3	11.7	9.4	11.4	14.8	14.8	5	4.7	19.2
SO_4 mg/dm^3	21	7	6	5	4	8	13	27	27	9	5	5
Clay	321	424	423	424	477	442	276.8	332	267	430	164	191
Sand	474	363	366	402	287	342	520.1	438	519	328	720	599
Silte	205	213	211	174	236	216	212	230	214	242	115	210

Legend: O. M. - Organic matter, pH - Hydrogen potential, S.B - Sum of bases, CTC - Cation exchange capacity, V - Base Saturation, m - Aluminium saturation, H+Al - Exchangeable acidity, P - Phosphor, K - Potassium, Ca - Calcium, Mg - Magnesium, Al - Aluminium, B - Boron, Cu - Copper, Fe - Iron, Zn - Zinc, Mn - Manganase, SO - Sulfates.

Table S2. Relative abundance of OTUs taxa (%) ≥ 0.5 found in plant tissues (roots and leaves) of *Aspillia grazielae* (Ascomycota Phylum).

Classes	Orders	Families	Genera	Roots		Leaves	
				NVA	RCA	NVA	RCA
Dothideomycetes	Capnodiales	Incertae sedis	*Toxicocladosporium*	*	*	0.67	0.06
		Mycosphaerellaceae	*Cladosporium*	0.88	0.97	0.94	1.47
			Mycosphaerella	*	*	1.34	0.84
			Passalora	*	*	0.59	0.56
	Dothideales	Dothioraceae	*Aureobasidium*	*	*	2.49	2.38
	Pleosporales	Pleosporaceae	*Curvularia*	*	*	0.59	0.07
			Leptosphaerulina	*	*	0.51	0.63
		Incertae sedis	*Phoma*	0.36	0.41	0.71	1.62
Eurotiomycetes	Eurotiales	Trichocomaceae	*Aspergillus*	0.44	0.62	0.33	0.21
			Eurotium	0.46	0.44	0.28	0.27
			Penicillium	10.17	4.45	5.97	4.88
Lecanoromycetes	Teloschistales	Teloschistaceae	*Xanthoria*	*	*	0.47	0.30
Sordariomycetes	Diaporthales	Valsaceae	*Phomopsis*	0.04	1.57	0.43	0.30
	Hypocreales	Hypocreaceae	*Trichoderma*	5.81	0.34	5.27	6.12
		Nectriaceae	*Fusarium*	2.05	2.08	1.14	1.12
	Xylariales	Amphisphaeriaceae	*Pestalotiopsis*	*	*	0.49	0.40

Legend: OTUs - operational taxonomic units; NVA - native vegetation area; RCA - recovery area (RCA); * - OTUs at genus level identified with Relative abundance ≤ 0.0001.
